# A Novel Aqueous Two Phase System Composed of Surfactant and Xylitol for the Purification of Lipase from Pumpkin (*Cucurbita moschata*) Seed sand Recycling of Phase Components

**DOI:** 10.3390/molecules200611184

**Published:** 2015-06-17

**Authors:** Mehrnoush Amid, Mohd Yazid Manap, Muhaini Hussin, Shuhaimi Mustafa

**Affiliations:** 1 Department of Food Technology, Faculty of Food Science and Technology, Universiti Putra Malaysia, 43400 UPM Serdang, Selangor, Malaysia; E-Mails: myazid@upm.edu.my (M.Y.M.); muhaini786@gmail.com (M.H.); 2 Halal Products Research Institute, Universiti Putra Malaysia, 43400 UPM Serdang, Selangor, Malaysia; E-Mail: shuhaimi@upm.edu.my; 3 Department of Microbiology, Faculty of Biotechnology and Biomolecular Science, Universiti Putra Malaysia, 43400 UPM Serdang, Selangor, Malaysia

**Keywords:** novel aqueous two phase system, surfactant, xylitol, recycling of phase components, lipase, *Cucurbita moschata*

## Abstract

Lipase is one of the more important enzymes used in various industries such as the food, detergent, pharmaceutical, textile, and pulp and paper sectors. A novel aqueous two-phase system composed of surfactant and xylitol was employed for the first time to purify lipase from *Cucurbita moschata*. The influence of different parameters such as type and concentration of surfactants, and the composition of the surfactant/xylitol mixtures on the partitioning behavior and recovery of lipase was investigated. Moreover, the effect of system pH and crude load on the degree of purification and yield of the purified lipase were studied. The results indicated that the lipase was partitioned into the top surfactant rich phase while the impurities partitioned into the bottom xylitol-rich phase using an aqueous two phase system composed of 24% (*w*/*w*) Triton X-100 and 20% (*w*/*w*) xylitol, at 56.2% of tie line length (TLL), (TTL is one of the important parameters in this study and it is determined from a bimodal curve in which the tie-line connects two nodes on the bimodal, that represent concentration of phase components in the top and bottom phases) and a crude load of 25% (*w*/*w*) at pH 8.0. Recovery and recycling of components was also measured in each successive step process. The enzyme was successfully recovered by the proposed method with a high purification factor of 16.4 and yield of 97.4% while over 97% of the phase components were also recovered and recycled. This study demonstrated that the proposed novel aqueous two phase system method is more efficient and economical than the traditional aqueous two phase system method for the purification and recovery of the valuable enzyme lipase.

## 1. Introduction

The aqueous two-phase system (ATPS) technique is an ideal method to separate and purify proteins; it is fast and economical and the processes are easy to implement because the clarification, concentration and partial purification of the target product can be carried out in one step [1]. This process is also easy to scale up, consumes little time and energy, has a low cost and ensures a high yields [2,3]. An ATPS is typically based on a polymer/salt system, such as PEG/potassium phosphate, or a polymer/polymer system, such as polyethylene glycol (PEG)/dextran [4]. The main drawbacks of these conventional systems are the fact that the phase-forming chemicals cannot be recycled effectively, which results in a large consumption of chemicals/polymers, high production costs and environmental pollution [5,6]. It has also been widely reported that additional, tedious purification operations, such as ultrafiltration, diafiltration and crystallization, are often needed to remove the phase-forming chemicals/polymers from the desired proteins recovered from these conventional ATPS [7,8]. To improve the traditional ATPS, a more economical and environmental friendly ATPS with the ability to retain the biological activity of enzymes is preferable compared to other conventional ATPS. The novel ATPS composed a surfactant and xylitol described herein overcomes this drawback of the traditional ATPS methods. This system makes it possible to create two phases and the surfactant rich top phase (*i.e*., containing non-ionic surfactants such as Triton X-100 and Tween 80, or an ionic surfactant like SDS) and the xylitol bottom phase can be recycled with high recovery of the purified enzyme [9]. The improved ATPS method can thus minimize the overall cost and the process of separating the target proteins from the two-phase solution will be simplified. Furthermore, recycling the solution components can minimize environmental pollution. Lipases are a class of hydrolytic enzymes which break triacylglycerols into glycerols and free fatty acids [10]. It has been reported that these enzymes account for 28% of the global enzyme sales [11]. Lipases have a wide range of applications in a variety of sector such as the food, biodiesel [12], cosmetic, pharmaceutical, leather, textile, detergent, and paper industries [13].

Pumpkin is a plant that has been widely used as functional food or medicine [14] and one of the important commercial tropical fruits in the world. Its production is increasing due to high demand as a healthy and nutritive table fruit [15]. The edible part of the pumpkin, which makes up 45%–80% of the fresh fruit, is processed into many products, while its seeds, which constitute 18%–20% of the whole fruit weight and contain valuable kinds of enzymes, are not currently utilized commercially but rather discarded as waste material [16]. Pumpkin seeds could be used as a valuable, economical and abundant source to produce natural enzymes such as lipase. Recently, lipase from *Cucurbita moschata* was purified using gel filtration chromatography [17]. In another study pumpkin seed oil lipase was extracted using supercritical fluid extraction [18]. Microwave-assisted aqueous enzymatic extraction has been employed for the extraction of lipase from pumpkin seeds [19]. An aqueous two phase system using a surfactant and sorbitol was employed to purify pectinase from guava peel [20], and an aqueous two phase system based on surfactant was used for partitioning amino acids [21]. A purification method using surfactant and salt also was employed for purification of protease from kesinai leaves [22]. An aqueous two phase system using a surfactant and salt was employed to purify lipase from *Burkholderia psuedomallei* [23].

The proposed aqueous two phase system (ATPS) method using a surfactant and sorbitol is novel and to date, there are no reports on the application of this method. In addition, until now there have been no literature reports on the purification of lipase from pumpkin seed (*Cucurbita moschata*) using an aqueous two phase surfactant/xylitol system. In the present study, the feasibility of recovering lipase from the novel surfactant/xylitol ATPS was studied. The effects of different parameters such as the concentrations of surfactant and xylitol, composition of surfactant/xylitol phases, crude load and pH on the partitioning efficiency of lipase in the ATPS were determined to obtain a high purification factor and high yield of lipase. In addition, the recovery and recycling of the surfactant and xylitol at each recycling step was also investigated.

## 2. Result and Discussion

### 2.1. The Effect of Phase Components on Lipase Stability

**Figure 1:**
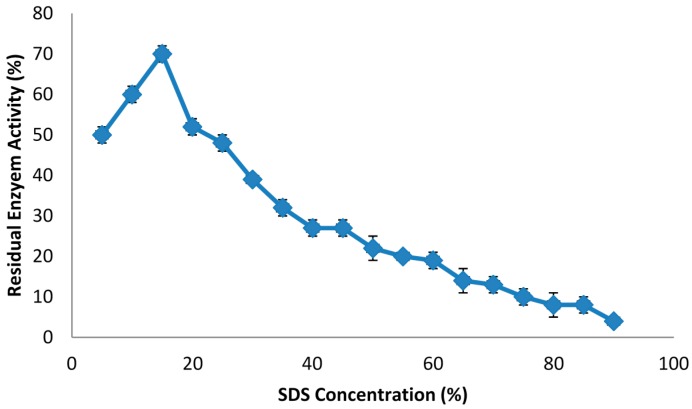
The effect of various concentrations of SDS on the lipase activity. The lipase was mixed with different concentrations of SDS from 5% to 90%. The activity of the enzyme in presence of SDS was then determined.

**Figure 2:**
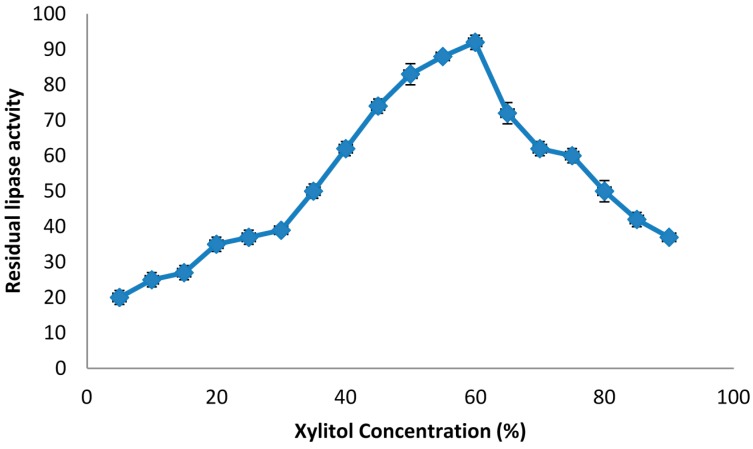
The effect of various concentrations of xylitol on the lipase activity. The lipase was mixed with different concentrations of xylitol from 5% to 90%. The activity of the enzyme in the presence of xylitol was then determined.

Preliminary studies revealed that the enzyme was stable in Triton X-100, Tween 80, SDS and xylitol, which indicated that the novel surfactant/xylitol-based ATPS could be suitable for the isolation of lipase. In order to assess the effect of each phase component on the lipase activity, crude lipase extract was mixed with different concentrations of the various compounds (Table 1). The increased activity of lipase from pumpkin seed promoted by a non-ionic surfactant (*i.e.*, Triton X-100) results in a decrease in the enzyme denaturation rate during hydrolysis [24]. Surfactants have also been reported to increase the availability of reaction sites and promote higher hydrolysis rates [25], therefore the non-ionic surfactant (*i.e.*, Triton X-100) acts as an inducer of the enzyme activity in the enzyme-substrate interaction. It would prevent inactivation of adsorbed enzymes and directly allows desorption of enzymes from substrate [26,27,28]. Table 1 shows that a high concentration (60%) of Triton X-100 and Tween 80 slightly activated the lipase activity. The effect of various concentrations of SDS on the lipase activity was also investigated. As shown in Figure 1, the lipase activity was increased by increasing of SDS concentrations up to 15%, while the activity was decreased by increasing of SDS concentration above 20% and SDS partially inactivated the enzyme. This latter effect was due to the binding of SDS, an ionic surfactant, to the proteins, which disrupted the majority of the globular proteins’ original structures. Further investigations were performed on lipase by mixing it with different concentration of xylitol to determine the lipase stability in that phase of the APTS. As the results in Table 1 indicate, lipase showed high stability in the presence of xylitol. In general, xylitol maintains the enzyme’s open conformation by exposing the active crevice surface site and finally stimulates the lipase activity [29]. Based on the results in Figure 2, the activity of the lipase increased as the xylitol concentrations increased from 5% to 60%, and the highest lipase activity was obtained in the presence of 60% xylitol. There is a rapid decrease in the activity with the increase of xylitol concentration above 65%, which caused a significant decrease on the lipase activity due to incompatibility of the denatured enzyme and substrate interaction.

**Table 1:** Effects of various phase compositions on the lipase activity of *Cucurbita moschata* Phase composition.

Phase Compositions	Concentration (% *w*/*w*)	Lipase Activity (U/mL)
Triton X-100	15	102.1 ± 0.23 ^a^
	30	110.1 ± 0.22 ^a^
	45	118.4 ± 0.11 ^b^
	60	142.2 ± 0.01 ^c^
	75	103.2 ± 0.01 ^d^
Tween 80	15	82.3 ± 0.11 ^a^
	30	81.1 ± 0.21 ^b^
	45	88.4 ± 0.22 ^c^
	60	95.6 ± 0.57 ^ab^
	75	92.3 ± 0.17 ^d^
SDS	15	52.3 ± 0.32 ^a^
	30	41.2 ± 1.21 ^b^
	45	38.2 ± 0.03 ^a^
	60	32.1 ± 0.32 ^a^
	75	27.2 ± 0.01 ^a^
Xylitol	15	132.1 ± 0.07 ^a^
	30	112.6 ± 1.10 ^b^
	45	98.1 ± 0.22 ^ab^
	60	92.3 ± 0.52 ^c^
	75	84.2 ± 0.12 ^d^

The crude lipase feedstock was incubated at room temperature for 1 h in each of the phase compositions. The residual activity was measured using a lipase assay. The lipase activity of Tris-HCL buffer (50 mM, pH 8.0) was used as the control. Each experiment was performed in triplicate. The results are expressed as the mean of triplicate readings, which have an estimated error of ± 5%. ^a–d^ Mean values followed by different letters differ significantly (*p* < 0.05).

Therefore, it is concluded that high concentrations of xylitol denature the lipase and decrease its activity compared to lower concentrations of xylitol. Ooi *et al*. [23] reported that the activity of the lipase derived from *Burkholderia pseudomallei* using an alcohol/salt aqueous two phase system decreased at high concentrations of the alcohol. In addition, they have reported that the high concentrations of salt also had a negative effect on the activity of the lipase from *Burkholderia pseudomallei*.

### 2.2. Selection of the Optimal Surfactants/Xylitol ATPS for the Lipase Partitioning

Table 2 summarizes the selectivity and purification factors of the lipase obtained with the different types of surfactants and xylitol. It is important to note that the effect of surfactants on the enzyme partitioning depends on selective chemical interactions between the enzyme molecules and surfactant. This effect is further divided into two factors which are: (i) structure of the enzyme and (ii) the chemical properties of the surfactant [30]. The results in Table 2 indicate that the selectivity and purification factors of the lipase in a non-ionic surfactant/xylitol system are significantly higher (*p* < 0.05) compared to an ionic ATPS. This could be due to the presence of protective amino acid surface loops acting as “lids” that cover the active site of the enzyme in its “closed” form [31,32]. A study has reported that the presence of non-ionic surfactants causes the lid to undergo a conformational rearrangement thus exposing the active site and forming the active, “open” form of the enzyme [26]. This conformational change of the lid in the presence of non-ionic surfactants could be a reason for the enhanced lipase activity and enzyme partitioning. The results showed that Triton X-100 increased the enzyme partitioning in comparison to Tween 80 and SDS had the lowest effect on the enzyme partitioning (Table 2).

**Table 2:** Partition behavior of lipase in different surfactant/xylitol systems.

System	Concentration of Surfactant/Xylitol (% *w*/*w*)	TLL (% *w*/*w*)	Selectivity	Purification Factor
TritonX-100/xylitol	15/17	54.3	51.01 ± 0.2 ^a^	7.31 ± 1.1 ^a^
	22/25	56.2	88.11 ± 0.1 ^b^	10.09 ± 1.1 ^b^
	26/28	59.1	66.15 ± 0.3 ^c^	8.12 ± 0.3 ^c^
	32/30	63.4	31.01 ± 1.1 ^d^	7.84 ± 0.2 ^d^
Tween-80/xylitol	17/18	22.4	22.20 ± 1.1 ^e^	3.33 ± 1.1 ^ab^
	22/20	33.3	32.12 ± 0.2 ^e^	3.12 ± 1.1 ^e^
	24/22	42.4	22.11 ± 0.1 ^ed^	2.81 ± 0.1 ^d^
	29/25	47.2	18.11 ± 0.3 ^e^	1.62 ± 0.3 ^e^
SDS/xylitol	13/12	33.2	12.23 ± 1.1 ^j^	2.12 ± 0.4 ^g^
	20/18	35.4	9.15 ± 2.3 ^k^	1.07 ± 0.2 ^h^
	22/19	46.1	7.11 ± 1.1 ^k^	0.72 ± 0.3 ^i^
	27/25	52.4	5.42 ± 0.2 ^jk^	0.34 ± 1.1 ^j^

The table summarises the partition achieved in phase systems composed of different surfactants (Triton X-100, Tween 80 and SDS) and xylitol solutions. The selectivity and purification factor were determined according to Equations (2) and (5), respectively. ^a–k^ Mean values followed by different letters differ significantly (*p* < 0.05).

It was discussed earlier that the binding of ionic surfactants to the protein would possibly cleave the protein tertiary structure. It has been verified that the ionic surfactant and the bonded proteins are joined together by electrostatic and hydrophobic forces [33,34]. Thus, the surfactant head group plays a determining role in the protein-surfactant interactions, which preferentially begin with the formation of strong ionic bonds between the surfactant polar groups [35]. This would then inhibit the enzyme partitioning in the system and eventually reduce the enzyme purification factor. In this section of experiment, the maximum selectivity achieved was 88.11 with a purification factor of 10.09 in the Triton X-100/xylitol ATPS system and hence this system was chosen for further optimization of surfactant/xylitol ATPS. Twenty systems were assessed to optimize lipase partition efficiency in a Triton X-100/xylitol system. Table 3 shows that the optimum condition for lipase partitioning involved 24% (*w*/*v*) Triton X-100 and 20% (*w*/*v*) xylitol, which resulted in a purification factor of 12.3 and a yield of 93.1%. Based on this result, it can be deduced that lipase partitioning performed well at low concentrations of surfactants and xylitol. High concentrations of surfactants negatively affected the amount of solubilized enzyme and its catalytic action. Similar effects were observed in xylitol when the concentration was increased, and these effects were likely due to the gradual dehydration of the bottom phase as the concentration of non-ionic surfactant in the top phase increased, which lead to an imbalance in the lipase retention in the top phase [36]. Shahbaz, *et al.* [37] reported that the purification of partitioning of amino acids in the presence of high concentrations of Triton X-100 was decreased in aqueous two phase systems. They have also reported that the stability of amino acids significantly decreased in the presence of the high concentrations of surfactants. 

**Table 3:** Partition of lipase in different concentrations of Tritonx-100/xylitol systems.

Triton X-100 (% *w*/*w*)	Xylitol (% *w*/*w*)	Purification Factor	Yield (%)
22	18	3.11 ± 0.2 ^a^	75.3 ± 0.2 ^a^
22	20	4.12 ± 1.1 ^b^	66.6 ± 0.3 ^b^
22	22	5.21 ± 0.1 ^c^	59.4 ± 10 ^c^
22	24	5.13 ± 0.2 ^d^	48.3 ± 1.3 ^ab^
22	26	5.10 ± 1.1 ^e^	43.3 ± 1.2 ^d^
23	18	6.01 ± 0.1 ^f^	32.6 ± 0.2 ^e^
23	20	6.13 ± 1.1 ^g^	23.4 ± 0.1 ^f^
23	22	6.42 ± 1.1 ^g^	21.2 ± 1.3 ^g^
23	24	7.72 ± 0.2 ^h^	18.3 ± 0.1 ^g^
23	26	7.81 ± 0.1 ^b^	10.6 ± 0.2 ^e^
24	18	8.83 ± 1.1 ^ab^	68.1 ± 0.1 ^f^
24	20	12.28 ± 0.1 ^i^	93.1 ± 0.1 ^g^
24	22	10.32 ± 0.2 ^i^	82.2 ± 0.1 ^h^
24	24	10.11 ± 0.1 ^i^	64.3 ± 0.2 ^i^
24	26	9.09 ± 0.2 ^i^	53.2 ± 1.1 ^j^
25	18	8.23 ± 0.1 ^j^	31.3 ± 1.1 ^k^
25	20	4.21 ± 1.1 ^i^	28.3 ± 0.3 ^k^
25	22	3.32 ± 0.2 ^k^	22.6 ± 1.2 ^j^
25	24	2.12 ± 0.3 ^l^	18.4 ± 1.0 ^l^
25	26	1.01 ± 2.1 ^m^	14.3 ± 0.2 ^m^

The partition behaviour of lipase in different concentrations of Tritonx-100/xylitol. The purification factor and yield of lipase were determined according to Equations (5) and (6), respectively. Each experiment was performed in triplicate. The results are expressed as the mean of triplicate readings, which have an estimated error of ±10%; ^a–m^ Mean values followed by different letters differ significantly (*p* < 0.05).

### 2.3. The Effect of Crude Feedstock Concentration on Lipase Partitioning

**Figure 3:**
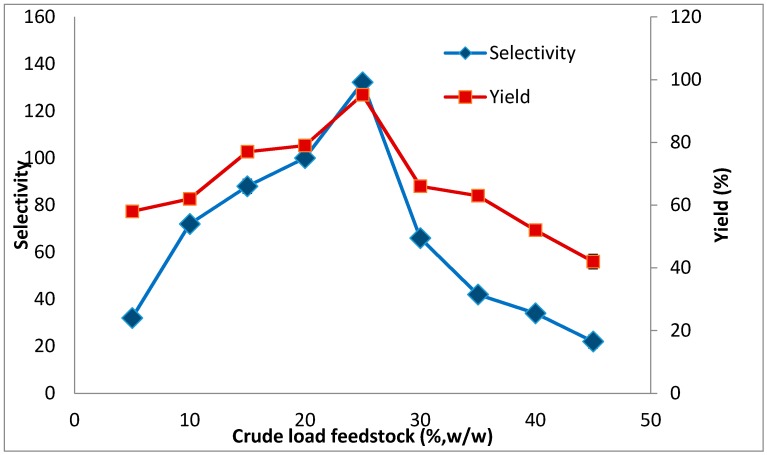
Influence of crude load on the partitioning of lipase. The selectivity (♦) and yield (■) were calculated as a function of the crude load, according to Equations (2) and (6), respectively. The results were expressed as the mean of triplicate readings, which have an estimated error of ±10%.

The increase in crude load is an advantage of the recovery processes using the ATPS technique. In fact, the effect of an increase in loaded crude on enzyme partition is important as it increases the phase volume ratio [38] and alters the partition behavior of the target proteins [39]. However, a high amount of lipase and its contaminants in the system would reduce the ATPS performance. This effect was evaluated by varying the amount of crude load up to 50% (*w*/*v*). The results depicted in Figure 3 show the effect of crude load on lipase recovery, indicating that 25% (*w*/*v*) of crude load yielded the maximum capacity per 10 g ATPS. The selectivity and the yield achieved by the 25% (*w*/*v*) crude load were 132.2% and 95.2%, respectively. The composition and volume ratio of the ATPS were greatly reduced by the loading of large amounts of sample into the ATPS. It should be noted that the components in the crude stock could alter the physical properties of ATPS and thus, ATPS with high concentration of crude load was unable to provide optimum purification conditions for the particular enzyme. Such behaviour can be explained by the increasing accumulation of precipitate at the interface, showing the loss of lipase together with other contaminants in the purification. The optimum conditions for purification of amylase from pitaya peel using an aqueous two phase system based on a thermo-separating polymer and an organic solvent were obtained in the presence of 25% of crude feedstock [40]. Show *et al.*, [41] reported that 20% of crude feedstock was suitable for purification of lipase from *Burkholderia cenocepacia* ST8. Based on our results 25% of sample loading would be appropriate for recovery of lipase from crude extract using Triton X-100/xylitol.

### 2.4. The Effect of System pH on Lipase Partitioning

**Figure 4:**
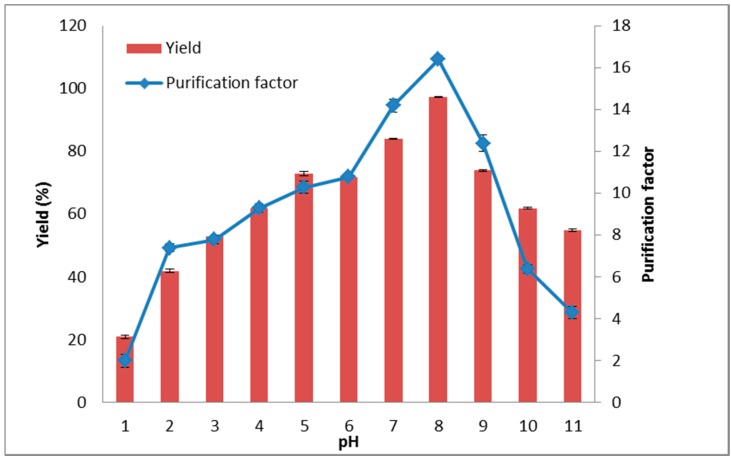
Influence of pH on partitioning of the lipase in Triton X-100/xylitol ATPS. Effect of various pHs on the partitioning of lipase into the top phase was investigated. The purification factor (♦) and yield (▬) were determined according to Equations (5) and (6), respectively.

The lipase partitioning in ATPS at different pH values is depicted in Figure 4 In general, the partitioning of biomolecules in ATPS is affected by pH that shifts the partitioning behavior of proteins by altering the target protein charge [42]. Furthermore, the manipulation of pH in the system could be correlated with electrochemical interactions between protein and solvent in the system [43]. It should be noted that the enzyme has an isolectric point (pI) of 8.03, hence at pH 8.0 it is prone to be negatively charged and partition depends on the surface properties instead of the net charge. Lipase is a negatively charged molecule favored to partition into the hydrophobic region. However, the partitioning direction differed for the target enzyme, which tended to partition into the more hydrophobic surfactant enriched top phase. The shift in partition behavior of lipase is due its protein charge. This effect has been assumed responsible for the low purification factor and yield of lipase when the pH is lower than 8.0. This result was due to decreases in lipase activity at pHs lower than 8.0 because the enzyme is in an active state and stable in alkaline pHs, and its activity might have decreased in the presence of neutral or acidic pHs. This could be due to internal electrostatic repulsion or loss of internal electrostatic attraction of the charges on the side chains of amino acid to which proteins opens up resulting in a decrease of enzyme activity. Thus, the maximum purification factor was 16.4, and the enzyme yield was 97.4% at pH 8.0 and hence this pH was selected as the optimum pH for this study A similar observation was made by Shahbaz *et al.* [37] who utilised purified recombinant phenylalanine dehydrogenase in the ATPS system. There was a decrease in pectinase partitioning from guava peel in the high pH value (pH > 8.0) in the aqueous two-phase system using surfactant and sorbitol [20]. A similar observation during protease purification from *Streptomyces* sp. was reported by Silva *et al.* [44]. They also reported that an acidic pH was not suitable for the enzyme and the optimum pH for the purification of the enzyme was pH 8.0.

### 2.5. Recycling of Phase Components

The novel ATPS has an important industrial advantage in that both phase components can be recycled with a high percentage of recovery of up to 97% and up to five recycle runs could be performed. Table 4 shows that only minor losses of the surfactant and xylitol occurred in the recycling steps and the new protein that is dispensed into the ATPS can still be partitioned into the surfactant-rich top phase. It should be noted that there was a significant decrease in the recovery of surfactant and xylitol after the fifth cycle. Therefore, the recovery of the components was stopped at cycle five.

**Table 4:** The recycle recovery of surfactant and xylitol systems.

System	Initial	Recycle Systems						
		First	Second	Third	Forth	Fifth	Sixth	Seventh
Recovery of surfactant (%)	99.4	98.8 ± 0.02	98.5 ± 1.1	98.1 ± 0.21	97.8 ± 0.06	97.3 ± 0.05	83.2 ± 1.15	62.2 ± 1.11
Recovery of xylitol (%)	99.3	99.1 ± 0.03	98.6 ± 0.2	97.8 ± 0.01	97.2 ± 0.13	97.0 ± 0.11	88.2 ± 0.12	78.4 ± 0.11

The concentrations of the surfactant and xylitol recovered were measured by refractive index using a refractometer. The recovery of surfactant and xylitol were determined for the each recycle and up to the seventh recycling step.

### 2.6. The Effect of Temperature on Lipase Activity

**Figure 5:**
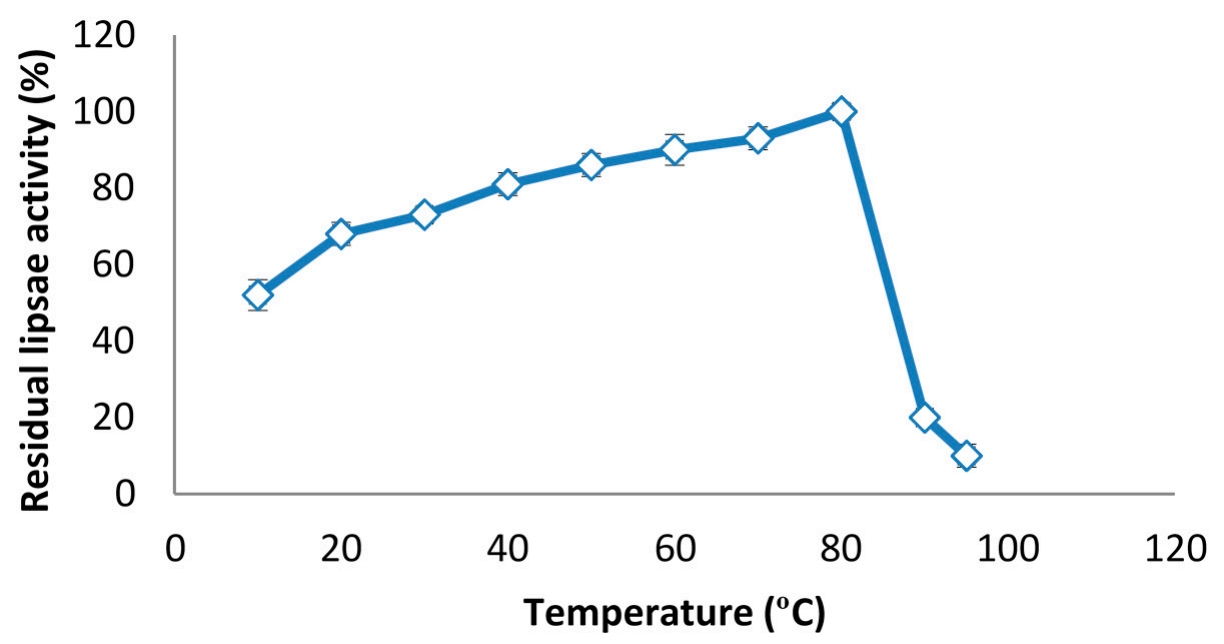
Effect of different temperatures on the enzyme activity of lipase from pumpkin seed. The residual lipase activity was determined after incubation of lipase at different temperatures.

The results presented in Figure 5 shows the effect of temperature on lipase activity from 10 to 95 °C in a 50 mM Tris-HCL (pH 8.0) solution. As shown, the activity of the lipase increased as the reaction temperature increased (from 10 to 80 °C) with the highest activity being observed at 80 °C. There was a rapid decrease in the activity with further increase of the reaction temperature beyond 88 °C. The rate constant of the thermal stability was determined by comparing the enzyme activity changes of the enzyme extract with and without heat treatment.

### 2.7. Lipase Recovery 

**Figure 6:**
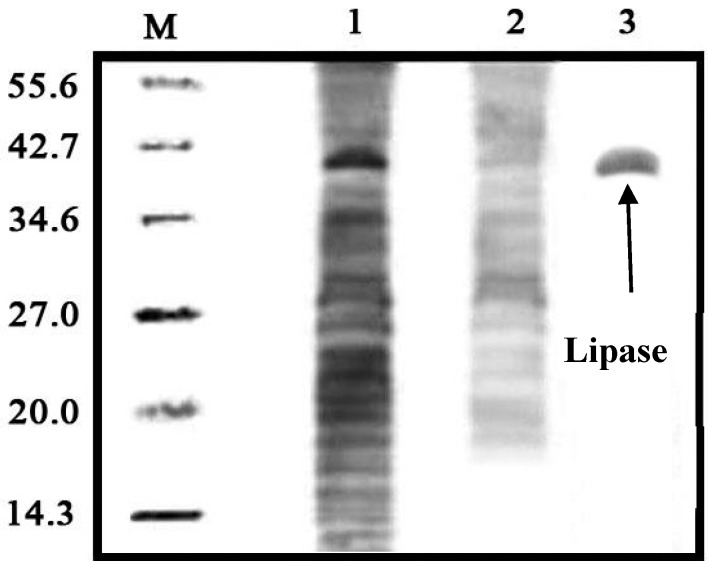
SDS-PAGE analysis of the lipase recovery. The protein molecular weight of the standard protein markers ranged from 6.5 to 55.6 kDa. Lines: M = protein molecular markers; 1 = crude feedstock; 2 = ATPS top phase lane; 3 = ATPS bottom phase.

The optimum conditions for lipase recovery in the Triton X-100/xylitol ATPS occurred for a tie line length (TLL) of 56.2% (*w*/*v*), and 25% (*w*/*v*) crude load at pH 8.0. The purity of lipase from pumpkin seed was determined via 12% SDS-PAGE and recorded in Figure 6 Lane 1 refers the crude feedstock sample with numerous impurity bands. Lane 2 is the bottom phase sample which was showed lesser and fainter bands. Finally Lane 3 corresponds to a recovered sample from the top phase, where there is only one dark band with a molecular weight of 39.2kDa. Hence, SDS-PAGE was able to confirm the presence of lipase in one of the phases.

## 3. Experimental Section

### 3.1. Materials

In this experiment, the chemicals and reagent used were analytical grade. The chemicals obtained from Sigma Chemical Co. (St Louis, MI, USA) were Bradford reagent, 4-nitrophenyl palmitate (pNPP) lipase substrate, bovine serum albumin (BSA), arabic gum powder and BioUltra Trizma^®^ base (Tris-Base) The other chemicals used were obtained from Merck (Darmstadt, Germany), namely Triton X-100, Tween 80, sodium dodecyl sulphate (SDS), acetic acid, sodium citrate, citric acid and hydrochloric acid. Pumpkins (*Cucurbita moschata*) at the same stage of ripening and with no visual defects were purchased from a local market; they were selected based on their size uniformity. Pumpkins were stored at 4 °C in a cold room before performing the extraction protocols.

### 3.2. Lipase Extraction from Pumpkin Seed

One hundred grams of fresh seeds of pumpkin fruit (*Cucurbita moschata*) were ground in a mixer for 2 min at room temperature in Tris-HCL buffer. The mixture was filtered through Whatman No. 4 filter paper to remove the precipitate, and the filtrate was collected as a crude extract for further processing. The ultrasound-assisted process of lipase extraction from *Cucurbita moschata* seeds was performed using an ultrasonic device (Elma S 30 H, Elmasonic, Luckenwalde, Germany) with a piezoelectric transducer connected to the frequency generator (37 kHz). A Schott bottle was held in the ultrasound processor to extract lipase for 5 min at 1:6 g/mL sample to solvent ratio with Tris-HCL buffer (pH 8.0) at room temperature. After extraction, the crude extracts were filtered and then centrifuged at 5000 rpm for 10 min. The feedstock was kept in a refrigerator at 4 °C for further use.

### 3.3. ATPS Composed of Non-Ionic Surfactant and Xylitol

The ATPSs for the purification of lipase from *Cucurbita moschata* were prepared in graduated glass centrifuge tubes after weighing the appropriate amounts of each surfactant (Table 2 and Table 3), the xylitol and the crude feedstock to reach a concentration of 20% (*w*/*w*) in the system. Deionized water was added to the mixtures to achieve a final mass of 10 g. The system was centrifuged at 4000 g for 10 min after mixing the components. After the two phases had become clear and transparent, and the interface was well defined, both phases were carefully separated. A long needle syringe was used to remove the bottom phase and a pipette was used to remove the top phase. The total volumes of the phases were measured, respectively. Subsequently, the samples were then analysed by a lipase activity assay and the aliquots were taken for determination of total protein content by Bradford analysis. Interference was avoided by preparing a tube with the same phase-forming component but in the absence of feedstock.

### 3.4. Lipase Assay and Protein Determination

The supernatant was assayed using *p*-nitrophenyl palmitate (pNPPa) as substrate to measure the activity of the extracted lipase. Supernatant sample (0.1 mL) was mixed with pNPP solution (0.9 mL) which contained pNPP (3 mg) in propan-2-ol (1 mL) diluted in 50 mM Tris-HCL buffer. The buffer was maintained at pH 8.0 with 40 mg of Triton X-100 and 10 mg of acacia gum powder. All these prepared materials were thoroughly mixed [36]. After 30 min incubation at 80 °C in a water bath, the absorbance was measured at 410 nm against an enzyme-free control using a spectrophotometer (BioMate™-3, Thermo Scientific, Alpha Numerix, Webster, NY, USA). One unit (U) of lipase activity was defined as the amount of enzyme required to convert 1 μmol of pNPP to *p*-nitrophenol per minute under specific conditions. Enzyme activity is expressed by U/mL [45]. The lipase assays were carried out triplicate and the averages were calculated. Protein concentration measurements were carried out using the Bradford assay [46] with bovine serum albumin (BSA) as standard.

### 3.5. Determination of Optimum Temperature of Lipase

The optimum temperature for lipase activity was studied by incubating it in a 50 mM Tris-HCL buffer (pH 8.0) within a temperature range of 10 to 95 °C. At different intervals of 2, 5, 10, 20, 30, 40, 60 min, samples were removed and the residual lipase activity was measured to determine the enzyme activity in the various temperatures [43].

### 3.6. Determination of the Enzyme Partitioning 

The partition coefficient (K) of the lipase was calculated as the ratio of the lipase activity in the two phases (Equation (1)):

(1) $$K=\frac{A_{T}}{A_{B}}$$

where *A_T_* and *A_B_* are the lipase activities in units/mL in the top and bottom phases, respectively. The specific activity (SA) was defined as the ratio between the enzyme activity (U) in the phase sample and the total protein concentration (mg) (Equation (2)):

(2) $$SA(U/mg)=\frac{Enzyme\;activity\;(U)}{\left[Protein\right](mg)}$$

The selectivity (S) was defined as the ratio of the lipase enzyme partition coefficient (*K_e_*) to the protein partition coefficient (K_p_) (Equation (3)):

(3) $$S=\frac{K_{e}}{K_{p}}$$

The volume ratio (*V_R_*) was defined as the ratio of volume in the top phase (*V_T_*) to that in the bottom phase (*V_B_*) (Equation (4)):

(4) $$V_{R}=\frac{V_{T}}{V_{B}}$$

The purification-fold (*P_FT_*) was calculated as the ratio of the lipase specific activity in the top phase to the initial lipase specific activity in the crude extract (Equation (5)) [39]:

(5) $$P_{FT}=\frac{SA\;of\;phase\;sample}{SA\;of\;crude\;stock}$$

Yield of lipase in top phase was determined using (Equation (6)):

(6) $$Y_{T}\left(\%\right)=\frac{100}{1+(1/V_{R}\times K)}$$

where *K* is the partition coefficient and *V_R_* is the volume ratio [47].

### 3.7. Recycling of Phase Components

The xylitol-rich bottom phase from the primary lipase recovery process was collected and reused in subsequent ATPS made up for phase component recycling study. Meanwhile, distilled water was added in a ratio of 1:1 to dilute the top phase of the primary lipase recovery ATPS. This was followed by incubating the diluted top phase sample in a water bath at 65 °C for duration of 15 min to achieve its thermo-separation. Centrifugation of the diluted top phase sample at 4000 rpm for 10 min to achieve phase separation, after which the top surfactant phase and bottom xylitol phase were separately withdrawn to complete the first recycling of the phase components. The next step was to weigh the xylitol bottom phase and to record the weight and determine xylitol recovery. The aqueous top phase which contained the enzyme was used for the lipase assay and to determine the total protein concentration while the top phase enriched by surfactant and the quantity of the recovered surfactant were measured. These recycling steps were repeated until the total elimination of the optimized conditions for the partitioning of the lipase in the surfactant/xylitol ATPS was achieved. The concentrations of the surfactant recovered were measured by refractive index using a refractometer and the calculation of the recovered surfactant was carried out as shown in Equation (7) below [41]:

(7) $$R=\frac{{\text{M }}_{\text{components after separataion}}}{{\text{ M }}_{\text{initial}}}\times 100$$

where M _components after separation_ in indicates the mass of recovered xylitol and surfactant in the top and bottom phase respectively, after separation and M _initial_ is the total mass of each component in the system.

### 3.8. Electrophoresis

Sodium dodecyl sulfate polyacrylamide gel electrophoresis (SDS PAGE) was used with 6% stacking gel and 12% resolving gel to evaluate the samples from the crude extract, and the top and bottom phases in the ATPS. These samples were diluted in sample buffer and heated to boiling for 5 min and after that they were run at 50 V and 12 mA for 1 h. The samples were further stained using Coomassie Brilliant Blue R-250 and destained in a solution containing 40% (*v*/*v*) methanol and 10% (*v*/*v*) acetic acid to determine the desired protein bands [48].

### 3.9. Statistical Design and Analysis

All the samples were organized using a completely randomized design with three times replications and repeated twice for reproducibility. Significant differences between the mean values of the triplicate data were determined by Duncan’s multiple range test (DMRT) using one- way analysis of variance (ANOVA). The statistical differences were defined at *p* < 0.05.

## 4. Conclusions

In this study, the main factors evaluated are the effect of type and concentration of surfactants, xylitol concentration, crude load feedstock, and system pH on the partitioning behavior of lipase. The optimum conditions obtained are 24% (*w*/*v*) Triton X-100 and 20% (*w*/*v*) xylitol with 56.2% TLL (It has been considered in Table 2), 25% crude at pH 8.0. Using the optimized conditions, purified lipase enzyme was obtained in 97.4% yield with a purification factor of 16.4. Therefore, this study has demonstrated that the direct recovery of lipase from pumpkin waste via an ATPS based on a surfactant and xylitol is a potentially useful method for the purification of this enzyme from plant sources. It should be noted that the lipase purified from *Cucurbita moschata* could have various industrial applications in the food, detergent, pharmaceutical and textile industries. This novel ATPS method is eco-friendly because it uses a biodegradable surfactant and xylitol for lipase recovery. The highest recycling percentage that resulted from the use of these components was 97%, which indicates that this novel method of ATPS is a green technology that will help promote a cleaner environment. This study established a novel efficient and economical technology for the purification and recovery of lipase from fruit waste in large-scale production.

## Abbreviations

Aqueous two-phase system (ATPS); Bovine serum albumin (BSA); Duncan’s multiple range test (DMRT); Nitrophenyl palmitate (pNPP);One-way analysis of variance (ANOVA); Polyethylene glycol (PEG): Purification Fold (P_F_); Specific activity (SA); Sodium dodecyl sulfate polyacrylamide gel electrophoresis (SDS-PAGE); Tile Line length (TLL).
